# Omnidirectional Absorber by the Void Plasmon Effect in the Visible Region with Greatly Enhanced Localized Electric Field

**DOI:** 10.1186/s11671-019-2873-6

**Published:** 2019-02-05

**Authors:** Shiwei Shu, Chengping Huang, Meng Zhang, Yan Yan

**Affiliations:** 1 College of Electronic Science and Technology, Shenzhen University, Shenzhen, 518060 People’s Republic of China; 2 Key Laboratory of Optoelectronic Devices and Systems of Ministry of Education and Guangdong Province, College of Optoelectronic Engineering, Shenzhen University, Shenzhen, 518060 People’s Republic of China; 3 Department of Physics, Nanjing Tech University, Nanjing, 211816 People’s Republic of China

**Keywords:** Absorbers, Plasmonics, Fabry–Perot resonance, Void plasmons

## Abstract

We propose and investigate a wide-angle and high-efficiency absorber by using the void plasmon (VP) effect in a Fabry–Perot (FP)-like system, which consists of a perforated metal film and a ground metal plane separated by a dielectric spacer. A hybrid FP/VP resonance mode contributes to the high absorption efficiency. Besides the increased absorption, greatly enhanced localized electric-field intensity at “hot spots” (~ 2284 times) can be achieved. In addition, by varying the thickness of the perforated metal layer and the environmental refractive index, the position of resonance peak can be easily controlled. The proposed absorber can also work as a sensor for detecting the surrounding dielectric constant with the maximum value of the figure of merit (FOM) achieving 3.16 in theory. This work creates an alternative design for high-efficiency absorption devices.

## Background

Surface plasmon resonance (SPR), which is the coherent oscillations of electrons at the interfaces of noble metals and dielectric materials, is able to enhance the light absorption efficiency of noble metals [1]. Nowadays, the SPR-based absorbers have been widely researched with various plasmonic systems, including arrays of gratings [2–9], metallic nanoparticles [10–21], and nanoholes in metal films [22–25]. By changing the geometrical and physical parameters such as the shape, size, and material of structures as well as the dielectric environment, the absorption properties in the visible region can be effectively controlled and improved. In general, propagating surface plasmons (PSPs) and localized surface plasmons (LSPs) are belonging to SPR. Metallic nanoparticles are usually in company with the LSPs effect, while the perforations on the metallic film can induce both the PSPs effect and the void plasmon (VP) effect. The VPs are one type of LSPs associated with nanohole structures, which can sustain an electromagnetic dipole resonance akin to that of metallic nanoparticles [26, 27]. The PSPs effect in nanohole array-based absorbers can not only eliminate the drawbacks of polarization sensitivity in one-dimensional metallic grating-based absorbers, but also realize nearly perfect absorption at the same wavelength in the visible region using a larger feature size of nanopatterns compared to nanoparticle-array-based devices. Considering the above advantages, the absorption mechanism of the PSPs effect in nanohole array structures has been widely investigated and reported [22–25]. However, the PSP effect-induced absorption is very sensitive to the incident angle due to its inherent mechanism [28], which reduces the whole absorption efficiency in absorbers. In contrast, the VP effect-induced absorption is insensitive to the angle and the polarization of incident light. Meanwhile, as it is sensitive to the surrounding dielectric constant, the position of resonant absorption peak can be tuned via changing environmental materials, showing the potential for differentiating the refractive index of surrounding materials. Thus, a systematic study on the VP effect is very meaningful [25, 29–32]. Nonetheless, the VP-induced absorption efficiency is usually lower than that achieved with other effects, e.g., the Fabry–Perot (FP) effect in a metal-insulator-metal (MIM) structure.

In this paper, a wide-angle and highly efficient absorber, consisting of a perforated metal film and a ground metal plane separated with a dielectric layer, has been systematically studied. The combination and interplay of FP resonance in the spacer and the VP effect in the nanoholes give rise to absorption efficiency as high as 99.8%. Furthermore, the VP effect-induced absorption peak is controllable by modifying structural or physical parameters such as the perforated metallic film thickness, the period of the nanohole arrays, and the environmental refractive index. In addition, the position of the resonance wavelength is insensitive to the edge length of the square nanohole and the incident angle of light. It is worthy of mentioning that the proposed device could also work as a sensor detecting the environmental refractive index, where a figure of merit (FOM) of 3.16 (which is compatible with that of conventional metal nanoparticles [33, 34]) can be obtained. The results presented in this work could enlarge the scope of the absorption mechanism and may provide a new way for designing absorbers that have potential applications in such as solar cells, photodetectors, and thermal emitters.

## Methods

**Fig. 1:**
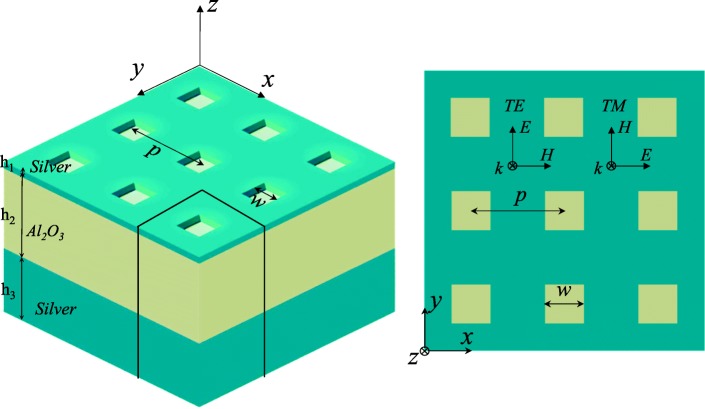
Schematic view of the proposed nanohole array-based absorber

The structure of designed absorber is schematically illustrated in Fig. 1, which contains a top silver layer milled with a square-hole array, an aluminum dioxide (Al_2_O_3_) middle layer, and a bottom silver layer. The thickness of each layer is denoted as *h*_1_, *h*_2_, and *h*_3_ respectively (*h*_3_ is assumed to be much larger than the skin depth of silver, thus preventing the transmission of light from the bottom silver layer). The period and edge length of the square holes in the top layer are denoted as *p* and *w*, respectively. The Lortenz–Drude model is used to describe optical constants of the silver [35]. The refractive index of Al_2_O_3_ is set as *n*_*d*_ = 1.76. The finite-difference time-domain (FDTD) method has been employed to investigate the optical properties of the structure. In all calculations, the simulation region was set as 200 × 200 × 2000 nm^3^ in three dimensions (where 200 nm is the lattice period). Period boundary conditions are set in *x*- and y-direction, and a perfectly matched layer (PML) is set in *z*-direction. A sufficient small mesh (1 × 1 × 1 nm^3^) is used in order to calculate absorption efficiencies and electric field distributions with high spatial resolutions. We set the simulation time as 1000 fs to make sure that the fields decay completely before the end of the simulation.

## Results and Discussion

**Fig. 2:**
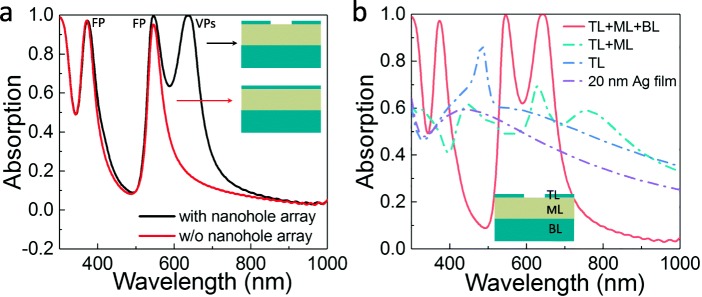
**a** Calculated absorption spectra of the proposed nanohole array-based absorber compared with the FP absorber without nanoholes in the top layer. **b** Calculated absorption spectra using different combinations of three layers as well as 20 nm of silver. TL, top layer; ML, middle layer; BL, bottom layer

Without loss of generality, the geometric parameters were set as *p* = 200 nm, *w* = 60 nm, *h*_1_ = 20 nm, *h*_2_ = 250 nm, and *h*_3_ = 200 nm. We suppose firstly that a plane wave is incident normally upon the structure. The black line in Fig. 2a presents the calculated absorption response. Compared with the FP absorber without the periodic nanohole arrays in the top layer, a new absorption peak around 635 nm appears with the absorption efficiency up to 99.8%. To understand the origin of this new absorption peak, the absorption spectra of different combinations of three layers together with a plane 20-nm-thick silver film are calculated and shown in Fig. 2b. Without the bottom reflection silver layer, the peaks corresponding to the FP resonance shift to the longer wavelength and present low absorption efficiency (see Fig. 2b; TL + ML), due to the variation of reflection phase at the bottom interface and the energy leakage via transmission. When the middle layer is removed, the FP resonance peaks dismiss eventually and the new absorption peak shows a large blue shift from 635 to 482 nm (see Fig. 2b; TL). The giant blue shift is related to the transition of the refractive index of environmental dielectric materials when the middle layer is removed. When the nanohole arrays are further removed (see Fig. 2b; a plane 20-nm-thick silver film), the sharp absorption peak around 482 nm disappears. Therefore, the new absorption peak located at 635 nm is correlated with the nanoholes in the top metal layer, where the peak position and absorption efficiency is modified by the coupling of VP resonance and FP resonance. The new peak is also sensitive to the refractive index of ambient materials, which provides a hint that it is related to the plasmonics effect (either PSPs or VPs). To further confirm the mechanism of the new absorption peak, numerical calculations are performed to analyze the possible PSPs mode of designed structures. It is shown that the maximal resonance wavelength for the silver/dielectric interface PSPs (0, 1) mode is 480 nm, which is much smaller than the resonance absorption peak at 635 nm. Therefore, we consider that the new peak originates from the VP effect of nanoholes.

**Fig. 3:**
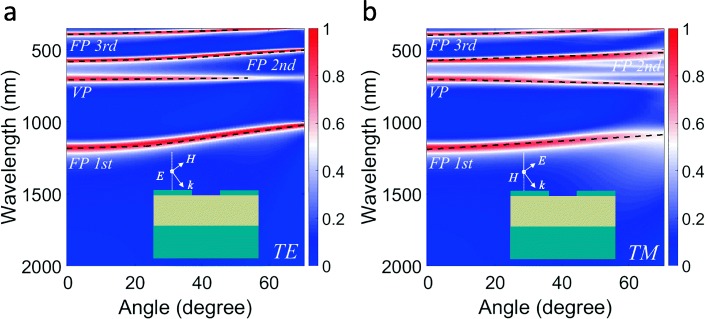
Calculated absorption contours of the proposed nanohole array-based absorber as a function of wavelength and incident angle: **a** TE and **b** TM polarization. Here, the structural parameters of the absorber are set as *p* = 200 nm, *w* = 60 nm, *h*_1_ = 20 nm, *h*_2_ = 250 nm, and *h*_3_ = 200 nm

The dependence of absorption peak position on the incident angles can further provide a strong evidence to distinguish the absorption mechanism between the PSPs and VP effect. To know the dispersion of the VP effect and strengthen our previous interpretation, we examine numerically the angle-dependent dispersion of the VP mode. The calculated absorption contours for the proposed absorber for transverse electric (TE) and transverse magnetic (TM) polarization are plotted respectively in Fig. 3a and b as a function of wavelength and incident angle. For TE polarization, as the incident angle *θ* increases, the VP absorption peak shows no shift, while the other three FP resonance absorption peaks shift toward the shorter wavelength. The peak shift of the FP resonance can be understood with the following resonance condition (the standing-wave condition in the middle dielectric layer):

1 $$ \left(4\pi {h}_2/\lambda \right)\sqrt{n_d^2-{\sin}^2\theta }+{\varphi}_1+{\varphi}_2=2\pi m, $$

where *φ*_1_ and *φ*_2_ are phase shifts at the top and bottom cavity interfaces and *m* is an integer. In addition, for TM polarization, the VP absorption peak shows a slight redshift as the incident angle rises. The three FP resonance absorption peaks present a blue shift, which is the same as that for TE polarization. In order to elucidate the absorption mechanism induced by VP resonance mode, we think the absorption induced by VP resonance mode includes two processes. The first process is the excitation of VP resonance mode induced by incident light. When the intrinsic resonance frequency of nanohole structure is the same as the frequency of incident light, the oscillation of conduction electrons at the interface is irrelevant to the polarization and angle of incident light. Then, the second process is the radiation of “resonance dipole” modulated by FP cavity. Because excitation and radiation are both independent on polarization and incident angle, the VP resonance mode induced absorption peak position is unchanged over the incident angle and the polarization.

**Fig. 4:**
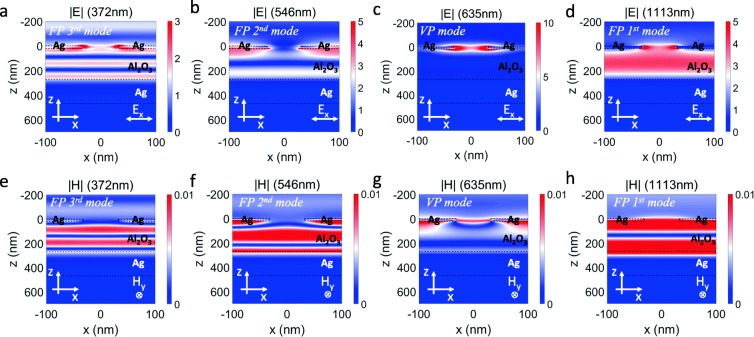
Calculated spatial field distributions of electric (**a**–**d**) and magnetic (**e**–**h**) fields for normal light incidence. The wavelength of incident light is 372 nm (**a**, **e**), 546 nm (**b**, **f**), 635 nm (**c**, **g**), and 1113 nm (**d**, **h**). The black dash lines denote the cross section of the structure. Here, the parameters of the structure are set as *p* = 200 nm, *w* = 60 nm, *h*_1_ = 20 nm, *h*_2_ = 250 nm, *h*_3_ = 200 nm, and *ε*_2_ = 3.1

The electromagnetic field distributions of the absorption peaks give more insights into the nature of absorption of FP and VP resonance. The calculated spatial electric field (upper panels) and magnetic field (lower panels) distribution for various absorption peak wavelengths are given in Fig. 4 (for normal incidence of light). For the FP resonance mode (372 nm, 546 nm, and 1113 nm), the electric field and magnetic fields are confined and boosted in the middle layer, and different resonance orders are formed corresponding to the specific patterns. With the electric and magnetic field pattern, it is observed that the first-order resonance mode locates at 1113 nm, the second-order resonance mode at 546 nm, and the third-order resonance mode at 372 nm. In contrast, for the VP mode at 635 nm, the electric field is greatly enhanced and localized on the edges of holes, as shown in Fig. 4c. Compared with the incident light, the maximal electric field intensity |*E*|^2^ of “hot spots” is enhanced by 2284 times. The strongly increased electric field intensity is beneficial for a large number of potential applications. Moreover, the magnetic field distribution demonstrates that the magnetic field is primarily confined near the top cavity interface, consistent with the localized character of the VP mode (see Fig. 4g).

**Fig. 5:**
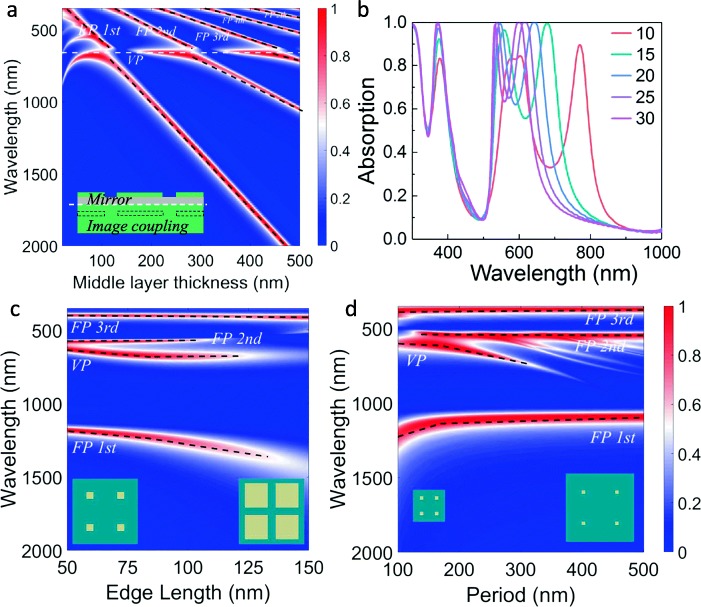
**a** Calculated absorption contour for the proposed nanohole array-based absorber as a function of the wavelength and the spacer layer thickness *h*_2_. The black dashed line represents the FP resonance and the white dashed line represents the VP mode. The inset shows the images (the dash rectangles) of the top metal layer with respect to the mirror metal interface (the white dash line). **b** Calculated absorption of the proposed nanohole array-based absorber dependent on *h*_1_ changing from 10 to 30 nm. **c** Calculated absorption contour for the proposed nanohole array-based absorber as a function of *w* with *p* = 200 nm. **d** Calculated absorption contour for the proposed nanohole array-based absorber as a function of *p* with *w* = 60 nm

In certain conditions, the FP and VP resonance may couple to each other, giving rise to a strong hybrid mode characteristic. To suggest the coupling between the FP and VP resonance, the dependence of absorption responses on the thickness *h*_2_ of the middle dielectric layer is studied by continuously tuning *h*_2_ from 20 to 500 nm. The results are depicted in Fig. 5a. As the thickness of the dielectric layer gets thicker, the wavelength of the FP resonance mode increases (the black dash lines), in agreement with the prediction of Eq. (1). Once the FP resonance wavelength overlaps the VP resonance wavelength (the white dash line), the FP and VP resonance modes are coupled into a hybrid FP-VP resonance mode. It is worthy of noting that the VP resonance mode may vanish when it is close to the FP resonance mode in certain conditions. In the absence of VP resonance mode, the strong absorption occurs at the FP cavity resonance, which also corresponds to the destructive interference between the light reflected from the top silver layer (with an extra half-wave loss of the phase) and that reflected from the bottom silver layer. When the wavelength of FP resonance mode approaches the VP resonance mode, the light is firstly absorbed by the nanohole structures, inducing a collective oscillation of conduction-band electrons near the silver nanoholes. Afterwards, as the oscillating dipoles, the nanoholes can emit radiations upward and downward. The upward light will interfere constructively with the reflected component of the downward light (reflected by the bottom silver layer). Thus, when the VP resonance mode coincides with the FP resonance mode, the destructive interference of the outgoing light may be transferred to the constructive interference. This scenario leads to a strong reflection and the absence of absorption in Fig. 5a (see the blue regions along the white dash line). It is also observed that when the dielectric layer thickness *h*_2_ is smaller than 50 nm, the VP-induced absorption efficiency is lower and the wavelength shows a redshift. When the dielectric thickness *h*_2_ is significantly reduced, the image of the VPs through the mirror metal interface will couple with the VPs of the top metal layer, leading to a decrease of mirror-coupling mode energy and an increase of resonance wavelength. The redshift of the absorption peak caused by the stronger mirror-coupling effect has also been proved by existing literatures [36, 37]. The absorption response of the proposed absorber for various thickness of the top metal layer thickness is also investigated, as presented in Fig. 5b. Clearly, the wavelength of absorption peaks caused by the resonance of the VP effect can be easily adjusted by altering the top layer thickness. As the top metal layer thickness *h*_1_ decreases, the absorption peak shows an obvious redshift, suggesting that the VP mode is susceptible to the thickness of the top layer. Furthermore, with the decrease of the top metal layer thickness, the second FP mode shows a slight redshift and the amplitude of absorption peak decreases gradually. This feature related to the second FP resonance mode is similar to that of the pure triple-layer absorber without nanohole arrays [38]. However, when the top layer thickness is reduced to *h*_1_ = 10 nm, there is apparent peak splitting (around 600 nm) which is not present in pure triple-layer absorbers.

The geometric effect of the nanoholes on the VP properties is also calculated. In Fig. 5c, the period of hole lattice *p* is fixed at 200 nm and the hole width *w* is changed from 50 to 150 nm. For FP absorption peaks, when *w* increases, the first-order mode resonance at 1113 nm shows a redshift, while the position of the second-order mode at 546 nm and the third-order mode at 372 nm almost remain unchanged. In addition, a redshift of the VP effect is also witnessed with the increase of *w*, as the electrons will experience a longer time when oscillating between two sides of the void (when the hole width *w* is larger enough, near-field coupling between two voids will be present as well [39]). In Fig. 5d, the effect of the lattice period on absorption properties of the VP effect is plotted. Here, *w* is fixed at 60 nm and *p* changes from 100 to 500 nm. For FP resonance absorption peaks, when *p* increases, the first-order resonance mode at 1113 nm shows a redshift when *p* is smaller than 200 nm and remains unchanged when *p* is larger than 200 nm. The redshift for the smaller *p* (*p* < 200 nm) is due to variation of effective medium refractive index of the top layer with *p* (or aspect ratio *w*^2^/*p*^2^). But, when *p* is larger than 200 nm, the effective medium refractive index is rarely affected by the small pore size. The second-order resonance mode at 546 nm and the third-order resonance mode at 372 nm show no shift when *p* changes. For the second FP mode, when *p* is larger than 300 nm, multiple emerged narrow absorption peaks will be present, which can be attributed to the PSPs effect. When the VP absorption peak (~ 635 nm) is concerned, a redshift is observed and the absorption efficiency becomes smaller as *p* grows. A similar phenomenon was also observed for absorbers based on the nanoparticle array and the redshift originates from a long-range dipole interaction [40]. Furthermore, we also find that the strong coupling of the VP resonance may inhibit the nearby FP effect. This phenomenon is observed in the situation where *w* is above 100 nm or *p* is smaller than 150 nm, as revealed in Fig. 5c and d. In general, a redshift of the VP absorption peak is in company with the increase of *w* or *p*.

**Fig. 6:**
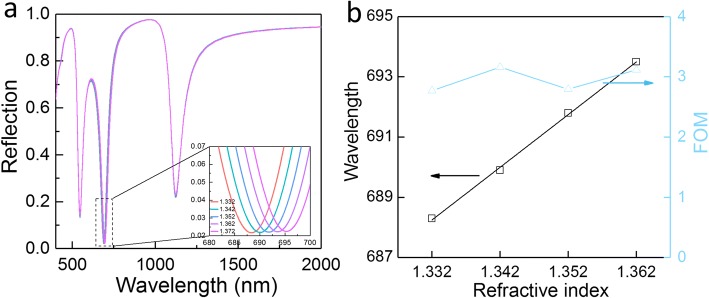
**a** Normal incidence reflection spectra of the proposed nanohole array-based absorber with the refractive index of hole (*n*) changing from 1.332 to 1.372. The structural and material parameters are set as *h*_1_ = 20 nm, *h*_2_ = 250 nm, *h*_3_ = 200 nm, *ε*_2_ = 3.1, *p* = 200 nm, and *w* = 60 nm. **b** The position of reflection dip and calculated FOM as a function of the refractive index of holes (*n* = 1.332–1.362)

Since the VP mode is confined near the nanoholes, the position of absorption peak induced by the VP effect is then dependent on the material refractive index in the holes. This effect can be employed to construct a sensor for distinguishing the surrounding dielectric constant. The reflection spectra for various material refractive indices in nanoholes have been calculated and plotted in Fig. 6a. The surrounding refractive index is changed from *n* = 1.332 (water) to *n* = 1.372 (minor glucose solution) with an interval of Δ*n* = 0.01. The FP resonance absorption peaks are almost irrelevant with the surrounding refractive index. On the contrary, just like LSPs characteristics, the VP absorption peak shows a dependence on the surrounding refractive index of the material. To measure the performance of a plasmonic sensor, a quantity called the figure of merit (FOM) can be used. The FOM is defined as sensitivity *S*_*λ*_ divided by linewidth *Γ*; here, *S*_*λ*_ is often simply denoted Δ*λ*/RIU (per unit change of refractive index) and *Γ* is the full width at half maximum (FWHM). In the calculation, we use a finer differential quotient with Δ*n* = 0.01 for *n* = 1.332, *n* = 1.342, *n* = 1.352, and *n* = 1.362. Figure 6b shows that the maximal sensitivity in terms of wavelength shift per refractive index unit is ≈ 186 nm/RIU. In our case, the resonance linewidth of VP mode is ≈ 59 nm and it leads to a maximal FOM ≈ 3.16. The FOM value in our work is compatible with reported devices based on metal nanoparticles [33, 34] (experimental FOM = 0.8–5.4) as well as the recently reported metal grating structures with theoretical FOM value of 2 [41]. However, it is much lower than the theoretical results achieved by highly complicated nanostructures [42, 43].

## Conclusions

In conclusion, we have systematically studied the VP effect in the nanohole-array-based tri-layer absorber using the FDTD method. By the VP effect, high absorption efficiency up to 99.8% and strongly boosted electric-field intensity (enhanced by 2284 times) can be achieved at the resonance wavelength. The high absorption efficiency is also benefited from the hybridization between the FP and VP mode. With the simulation, the intensity of the VP effect to light polarization and incident angle is proved, and the dependence of VP effect on the structural parameters is also investigated. Furthermore, the VP mode owns a maximal FOM value of 3.16, which may be useful for constructing the plasmonic sensors for detecting the environmental dielectric constant. The systematic study presented in this paper highlights the void of the absorption mechanism based on the VP effect and proposes a new design for high efficiency and multifunctional absorbers.

## Abbreviations

FDTD: Finite-difference time-domain
FOM: Figure of merit
FP: Fabry–Perot
LSPs: Localized surface plasmons
MIM: Metal-insulator-metal
PML: Perfectly matched layer
PSPs: Propagating surface plasmons
SPR: Surface plasmon resonance
TE: Transverse electric
TM: Transverse magnetic
VPs: Void plasmons
